# The negative compatibility effect with relevant masks: a case for automatic motor inhibition

**DOI:** 10.3389/fpsyg.2013.00822

**Published:** 2013-11-08

**Authors:** Brenda Ocampo, Matthew Finkbeiner

**Affiliations:** Department of Cognitive Science, ARC Centre of Excellence in Cognition and its Disorders, Macquarie UniversitySydney, NSW, Australia

**Keywords:** masked priming, positive and negative compatibility effect, unconscious processing, reaching trajectories, subliminal inhibition

## Abstract

For many years controversy has surrounded the so-called “negative compatibility effect” (NCE), a surprising phenomenon whereby responses to a target stimulus are delayed when the target is preceded by an unconscious, response-*compatible* prime. According to proponents of the “self-inhibition” hypothesis, the NCE occurs when a low-level self-inhibitory mechanism supresses early motor activations that are no longer supported by perceptual evidence. This account has been debated, however, by those who regard the NCE to be a stimulus-specific phenomenon that can be explained without recourse to a self-inhibitory mechanism. The present study used a novel reach-to-touch paradigm to test whether unconscious response priming would manifest as motor activation of the opposite-to-prime response (supporting mask-induced priming accounts), or motor inhibition of the primed response (supporting the notion of low-level self-inhibition). This paper presents new findings that show the emergence of positive and negative compatibility effects as they occur in stimulus processing time. In addition, evidence is provided suggesting that the NCE is *not* driven by the activation of the incorrect, “opposite-to-prime” response, but rather might reflect automatic motor inhibition.

## INTRODUCTION

The masked priming paradigm has been widely used to study the influence of unconscious information processing on behavior. Typically, a briefly presented visual “prime” stimulus is masked by a subsequent, spatially overlapping stimulus (the “mask”), such that conscious awareness of the prime is suppressed. In spite of this, responses to the subsequently presented target are faster (and more accurate) when the target is compatible with the prime, and slower (and less accurate) if the prime and target are incompatible (Neumann and Klotz, 1994; Ansorge et al., 1998; Leuthold and Kopp, 1998; Kiefer and Spitzer, 2000; Klinger et al., 2000; Damian, 2001; Jaskowski et al., 2002; Schmidt, 2002). This is known as the positive compatibility effect (PCE), and is believed to reflect the overlap in prime- and target-induced activation of motor pathways (Neumann and Klotz, 1994), and/or more abstract “semantic” representations (Finkbeiner and Friedman, 2011). Eimer and Schlaghecken (1998) reported an intriguing reversal of these effects when a delay is introduced between the prime and target. With interstimulus intervals exceeding ~100 ms, trials in which the prime and target are compatible produce slower responses than trials where the prime and target are incompatible (Eimer and Schlaghecken, 1998; Eimer, 1999; Schlaghecken and Eimer, 2000, 2001, 2002; Klapp and Hinkley, 2002). According to the self-inhibition account, this so-called negative compatibility effect (NCE) reflects a low-level and automatic process of inhibitory motor control (Schlaghecken et al., 2007).

**FIGURE 1:**
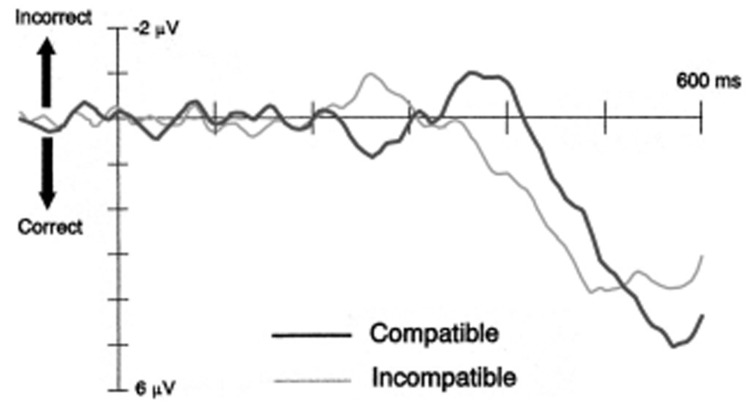
**Lateralised readiness potentials for compatible and incompatible trials from Experiment 2 of Eimer and Schlaghecken (1998)**.

The self-inhibition hypothesis was inferred from an early electrophysiological study showing a specific sequence of movement-related lateralized readiness potential (LRP) modulations (see **Figure 1**). Eimer and Schlaghecken (1998) observed that ~200 ms following prime onset, the LRP showed an initial tendency to prepare the response indicated by the prime. For compatible trials, this primed response tendency activated the correct response relative to the upcoming target, whereas for incompatible trials it overlapped with the incorrect response. Crucially, ~350 ms following prime onset the LRP signal reversed, resulting in the inhibition of the primed response and the disinhibition of the opposite response. Proponents of the self-inhibition account argue that primes initially trigger their assigned response and simultaneously inhibit the competing response alternative. This imbalance in response activation levels will produce a PCE if the target appears immediately after the prime. Importantly, because sensory evidence of the prime is suddenly removed by the mask, its associated response activation becomes obsolete. Therefore it becomes supressed, and the competing response is released from inhibition. If the target appears during this second phase, self-inhibition of the primed response (and disinhibition of its competitor) will produce an NCE (Schlaghecken and Eimer, 2006). This sequence of response activation followed by inhibition proved to be replicable (Eimer, 1999; Eimer and Schlaghecken, 2003; Verleger et al., 2004; Praamstra and Seiss, 2005), and is consistent with behavioral findings showing PCEs at short prime-target intervals, and NCEs at long prime-target intervals. Taken together this evidence has been used to build a case for unconsciously triggered inhibitory control.

This interpretation has attracted much attention, primarily because it challenges traditional views of control processes being volitional and hence dependent upon conscious awareness (Egner, 2010). Consequently, several alternative interpretations have been offered that, on the whole, provide an account for the NCE without invoking an automatic, self-inhibitory mechanism (Lleras and Enns, 2004, 2006; Verleger et al., 2004; Jaskowski and Przekoracka-Krawczyk, 2005; Jaskowski, 2008). Typically, the NCE is elicited in an experimental paradigm that employs arrows as the prime and target stimuli. These stimuli are presented at fixation, and the masks are constructed from potentially task-relevant elements (e.g., diagonal lines). According to mask-induced priming accounts, when a mask contains such “arrow-like” features these can interact with those of the preceding prime to produce a second prime that points in the opposite direction, and thereby primes the opposite response. Lleras and Enns (2004) and Verleger et al. (2004) claim that self-inhibition plays no role in the NCE. Instead, they suggest that the reversal of priming effects – from PCEs at short prime-target intervals to NCEs at long prime-target intervals – reflects a sequence of two positive priming events of opposite direction. The first of these is characterized by the initial, prime induced activation of the response associated with the prime. The second priming event occurs when the mask itself triggers the activation of the opposite response. These authors propose that the opposite peak of the LRP at 350 ms might therefore reflect the *activation* of the opposite response rather than the inhibition of the primed response. Although other explanations have been offered regarding the mechanisms underlying the NCE (c.f. Jaskowski, 2008), in the present study we will focus specifically on the predictions made by Lleras and Enns (2004) “object-updating” and Verleger et al. (2004) “active mask” hypotheses. Although these two accounts differ in several ways, they both emphasize that perceptual interactions between the prime and mask can produce inverse priming effects when both stimuli are comprised of common features. Henceforth, this account will be referred to as *mask-induced priming.*

The mask-induced priming account has received considerable support, with studies showing that the use of an irrelevant mask that does not contain arrow-like features substantially decreases (if not eliminates) the magnitude of the NCE (Lleras and Enns, 2004; Verleger et al., 2004; Jaskowski and Przekoracka-Krawczyk, 2005). Even though it was later shown that the NCE can indeed be obtained with irrelevant masks (Klapp, 2005; Schlaghecken and Eimer, 2006; Schlaghecken et al., 2007), it is now generally accepted that when a prime and mask *do* share common elements the NCE can be attributed to perceptual interactions between the two stimuli rather than self-inhibition (Klapp, 2005; Schlaghecken and Eimer, 2006). Just like the self-inhibition account, mask-induced priming predicts PCEs at short prime-target intervals and NCEs at long prime-target intervals. The difference between the two theories is in the mechanisms driving this effect. Specifically, at long prime-target intervals mask-induced priming predicts two instances of “positive” priming: the first triggered by the prime, and the second by its interaction with the mask.

The present study was designed to specifically test this prediction. In our view, the best way to determine that prime-mask interactions can produce the NCE would be to show that prime-mask similarity leads to the activation of the opposite-to-prime, incorrect response. However, because reaction times (RTs) reflect only the endpoint or culmination of target processing, we cannot conclude with certainty that slower RTs during compatible conditions are in fact caused by the activation of the incorrect response (rather than inhibition of the primed response). Nor can existing LRP findings shed light on the matter. In Eimer and Schlaghecken’s (1998) study, the upward-going (incorrect) LRP deflection observed for compatible trials in the 300–400 ms interval following prime onset cannot be (necessarily) interpreted as activation of the incorrect response (Eimer and Schlaghecken, 2003). As emphasized by the authors, LRPs reflect the relative, not the absolute, activation level of response tendencies and thus cannot reveal whether the incorrect response became selectively activated at any stage during processing. Although previous attempts have been made to shed light on this specific matter by comparing EEG signals between relevant and irrelevant masks (Verleger et al., 2004), and by combining transcranial magnetic stimulation (TMS) with motor evoked potentials (MEPs) (Verleger et al., 2006), due to various methodological issues these studies were unable to provide conclusive results (see General Discussion, Verleger et al., 2006). Therefore, the question remains whether, when a prime and mask share common elements, the empirical NCE is driven by an interaction between these two stimuli that results in the activation of the incorrect response.

Our aim was to test the directional claims of the mask-induced priming account using a novel methodological approach recently put forth by Finkbeiner et al. (2013). Rather than pressing a button to indicate their response, subjects in the present study classified the direction of a target arrow by reaching to touch the left or right side of the computer monitor. A motion capture device (Polhemus Lyberty) was used to sample the position of the hand during the reaching response, resulting in a high resolution continuous dataset on each trial. The use of such continuous movement measures in the cognitive psychology literature is growing (Spivey et al., 2005; Dale et al., 2007; Song and Nakayama, 2009; Chapman et al., 2010; Quek and Finkbeiner, 2013), primarily due to their purported ability to capture dynamic interactions between cognitive processes and motor output. The main advantage of reaching responses in this study is that they allowed us to quantify how quickly subjects moved their hand in the *correct* vs. incorrect direction upon perceiving the target stimulus. This was achieved by calculating “x-velocity” (described in further detail below), a signed value that is positive when subjects moved in the correct direction and negative when they moved in the incorrect direction. As such, x-velocity represents a much more informative measure than nominal accuracy rates or RTs, which range from “fast” to “slow” in a single positive direction (Finkbeiner et al., 2013).

In our experimental paradigm, we employed prime and mask stimuli that contained common arrow-like elements and we used a long prime-target stimulus-onset asynchrony (SOA) of 150 ms. Under these circumstances, the mask-induced priming account predicts that the newly emergent features contained within the mask will resemble an arrow pointing in the opposite direction to the prime. We therefore expected that on trials where the subsequent target was compatible with the prime, initial x-velocities would be negative, reflecting an early tendency for subjects to move in the opposite-to-prime, incorrect direction. If, however, x-velocities for compatible trials are simply *slower* than those for incompatible trials (but not negative), then we could not conclude with certainty that prime-mask interactions produce the NCE.

Using the reach-to-touch paradigm, we also set out to map the onset and growth of both PCEs and NCEs using a single prime-target interval. With this goal in mind, we asked subjects to initiate their movement within 300 ms of an imperative go signal, defined as the final beep in a sequence of three beeps. The final “go” beep was randomly positioned on each trial to occur at different time-points relative to the onset of the target. This allowed us to elicit movements that commenced across a wide set of *target-viewing times*, ranging from before target onset to ~400 ms thereafter. The time (in milliseconds) from target onset to movement onset will henceforth be referred to as movement initiation time (MIT). Based on LRP demonstrations of a bi-phasic activation-inhibition sequence, we reasoned that reaching movements initiated very early on during stimulus processing (i.e., at short MITs) should occur when the prime-evoked motor response is still active, and thus be characterized by a PCE. Conversely, at longer MITs initial primed activations should have been overridden by the mask, which in turn should activate the incorrect response. Here, we expected a NCE.

## MATERIALS AND METHODS

### PARTICIPANTS

Twenty volunteers (6 male) aged 18–25 years participated in the experiment. All were right-handed, and had normal or corrected-to-normal vision. One participant was excluded from further analysis because of excessive error rates (more than 20% errors). The Human Research Ethics Committee of Macquarie University approved the experiment, and written informed consent was obtained from all participants.

### STIMULI

**FIGURE 2:**
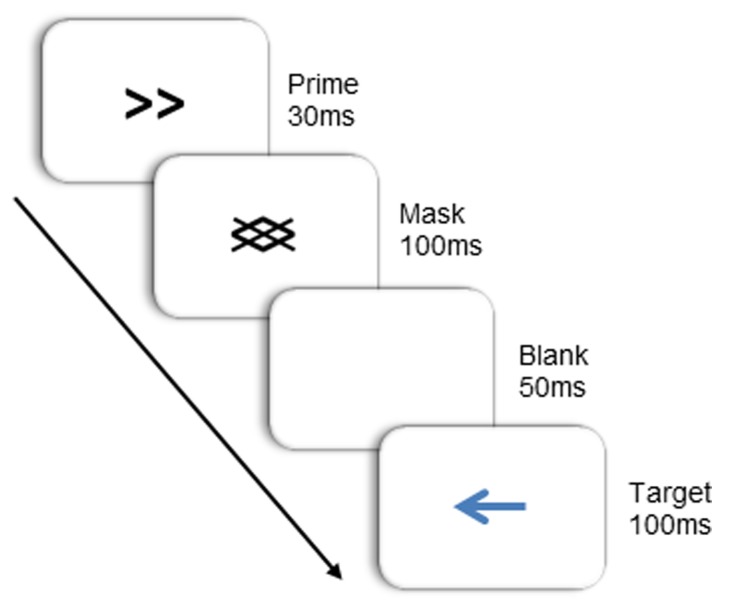
**Stimulus and trial-structure**.

Target stimuli were blue arrows pointing either left or right, subtending a visual angle of approximately 1.4° × 2.4°. Primes were identical in size, and consisted of double-headed arrows that also pointed right or left. Masks were composed of oblique lines of the same orientation as the lines in the prime stimuli (see **Figure 2**). Primes and masks appeared in black. All stimuli were presented on a white background at the center of the screen. Note that in this study, the target arrow was physically dissimilar to the prime arrow. This was done to limit the role of learned stimulus-response mappings (Finkbeiner and Friedman, 2011) and to better isolate the source of the NCE to the integration of the prime and backward mask.

### EQUIPMENT

The experiment was run on a Dell Optiplex GX990 running 64-bit Windows. A Samsung S27SA950 LED monitor was used at a resolution of 1920 × 1080, which allowed a screen refresh rate of 120 Hz. Stimulus presentation was controlled using Presentation software (Neurobehavioural Systems), and custom software was written to interface the stimulus display with the motion capture device (Pohlemus Liberty). This electromagnetic device recorded participants’ reaching trajectories by sampling the position of a small sensor taped to the tip of the right index finger at a rate of 240 Hz. Subjects wore headphones (Sennheiser 280 Pro) which were used to present the sequence of beeps.

### PROCEDURE

Subjects were seated in a dimly lit room, facing a computer screen at a viewing distance of 50 cm. Two lateral response boards (30 cm × 9 cm) were positioned on either side of the computer monitor, 75 cm apart and 50 cm from the table edge. Subjects initiated a trial by moving their right index finger to the “start” position, which was located in the middle of the desk and close to its front edge. Each trial began with a fixation dot, during which the sequence of beeps began. Then, the prime arrow was presented for 30 ms, followed immediately by the mask for 100 ms. After mask offset, the screen remained blank for 50 ms, then a target was presented for 100 ms. Primes were either compatible with the target (both pointing in the same direction), or incompatible (each pointing in opposite directions). To respond, subjects reached out and touched the left response board when the target arrow pointed to the left, and the right response board when it pointed to the right. Subjects initiated their responses when they heard the last of the three sequentially presented beeps, which either co-occurred with target onset (40% of the time; 0 ms SOA), or appeared sometime thereafter (SOAs of 150 or 250 ms, 40 and 20% of the time respectively). The response window opened 100 ms before the go signal (third beep) and closed 200 ms after the go signal. If movement was not initiated within this timeframe, the trial was terminated with a buzz and the appropriate visual feedback (e.g., “Too Early!” or “Too Late!”) was presented. Trials that were terminated due to a response window failure were re-presented at a later point in the experiment. Upon initiating a movement, subjects were required to maintain a continuous forward movement over the first 50 recorded samples (~208 ms) and trials were terminated with a buzz and visual feedback if this criterion was not met.

After a practice block of 20 trials, subjects completed a total of 360 experimental trials. At the conclusion of the experiment, we assessed subjects’ awareness of the primes by asking them to complete 140 trials of a two-alternative forced choice prime identification task. They were informed of the prime’s presence and, following classification of the target stimulus, they were instructed to indicate for each trial which of two arrows appeared as the prime by pressing a right or left button.

### DATA ANALYSIS AND RESULTS

Reaching trajectories were time-normalized prior to analysis by re-sampling each to produce 101 evenly spaced increments between the point corresponding to 10% of peak tangential velocity and the point at which the finger touched the response board. As a first step in this analysis, we applied a two-way lowpass Butterworth filter to the position data using a frequency of 7 Hz. Derivatives (velocity/acceleration) were then calculated through numerical differentiation. We employed two separate analyses to test whether (a) both PCEs and NCEs could be observed within a single experimental context, and (b) the NCE was characterized by movements in the incorrect direction (as predicted by the mask-induced priming account).

### PATHOFFSET

The first goal of our analysis was to establish that PCEs and NCEs could be elicited by varying target-viewing time. Ideally, we would compare reaching trajectories initiated purely on the basis of prime processing to those initiated following the processing of all (prime, mask and target) stimuli. As this is impossible to determine, we referred to the ERP data reported by Eimer and Schlaghecken (1998). Here, LRP waveforms indicated that the response assigned to the prime was elicited ~200 ms after prime onset. In our paradigm this corresponds to 50 ms following target onset. We reasoned that reaching movements initiated within 50 ms following target onset should reflect processing of the prime and thus show PCEs. Reaching movements initiated after 50 ms following target onset, on the other hand, should represent the “reversed polarity” found in LRP waveforms at around 300 ms post prime onset, and produce NCEs. Therefore, before computing the “pathoffset” analysis described below, we segregated our data based on how long subjects viewed the target for prior to initiating their response (MIT). Trials with MIT values <50 were classified as “pure prime trials,” and trials with MIT values >50 as “prime + target trials.”

Pathoffset, or “curvature,” is a measure referring to the perpendicular distance between the hand and the linear path connecting the first and last positions of the reaching movement (Finkbeiner et al., 2013). Typically, masked primes influence the finger’s initial flight path such that pathoffset values are larger in incompatible than in compatible prime conditions (Finkbeiner and Friedman, 2011). To get an overall impression of the effect of prime type on the reaching response, we computed pathoffset at each recorded sample, and then calculated the maximum pathoffset value for pure prime and prime + target trials. Based on the LRP findings mentioned above, we expected pathoffset values in pure prime trials to be larger for incompatible than for compatible prime conditions. Conversely, pathoffset for prime + target trials was expected to be smaller for incompatible than for compatible prime conditions.

Subjects’ accuracy rates are very high in the reach-to-touch paradigm. Presumably, this is due to the relatively long duration of the reaching response, which allows subjects to recognize and correct mistakes they may have made at the beginning of their movement. Therefore, accuracy rates were at ceiling (99.9% correct), and they were not modulated by prime-target compatibility.

**FIGURE 3:**
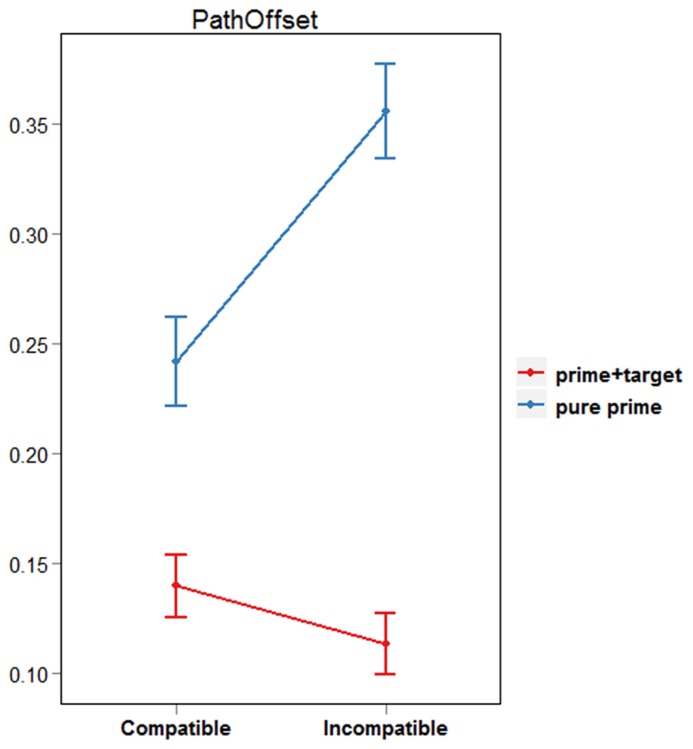
**Average pathoffset by prime type and trial type**.

Paired-samples *t*-tests were used to compare pathoffset values between compatible and incompatible trials. This was done separately for pure prime and prime + target trials because naturally, a smaller number of trials (10%) were initiated within 50 ms after target onset. Results revealed that pure prime trials were characterized by significantly larger pathoffset values when the prime and target were incompatible than compatible [*df*(18), *t* = -3.51, *p* < 0.01; see **Figure 3**]. The opposite result was found for prime+target trials, however, with larger pathoffset values for compatible compared with incompatible conditions [*df*(18), *t* = 2.57, *p* = 0.02]. These results support our claim that both PCEs and NCEs can be observed using a single prime-target interval and a continuous behavioral measure. Specifically, they are in line with our prediction that reaching movements initiated within 50 ms following target onset would reflect processing of the prime stimulus, whereas reaching movements initiated after 50 ms following target onset would represent the “reversed polarity” found in LRP waveforms ~300 ms post prime onset. However, it could be argued that the fewer number of trajectories in the pure prime condition resulted in insufficient power. To determine that trials with early MITs are indeed characterized by a PCE, we will refer to the results of our second analysis described below. Crucially, this analysis ensures an equal proportion of trials in each decile of the MIT distribution.

### INITIAL X-VELOCITY

The primary objective of this experiment, and the goal of this second analysis, was to investigate how the NCE would manifest in behavior during early stages of stimulus processing. This was achieved by employing a dependent measure that exploited the fact that subjects reached left for left arrows, and right for right arrows. X-velocity refers to the velocity of the hand as it moves along the task-relevant, left-right dimension. Because x-velocity is calculated so that it is positive for movements in the correct direction and negative for movements in the incorrect direction, it renders an analog value of the subjects’ ability to correctly classify the target stimulus. Compatibility effects with this measure are typically reflected in higher (more positive) x-velocities on compatible trials at earlier points in time (Quek and Finkbeiner, 2013). In our paradigm, we expect the *direction* of congruency effects to vary as a function of MIT. Recall that MIT is defined as the time from target onset until the participant released the start button and began their reaching response. Because MIT reflects the amount of time the participant had to accumulate evidence about the target prior to commencing their classification response, it serves as a proxy for target-viewing time. Therefore, trials with short MITs were predicted to show a PCE, with higher x-velocities during compatible vs. incompatible prime conditions. On the other hand, trials with longer MITs were expected to exhibit a NCE characterized by positive x-velocities during incompatible prime conditions, and negative x-velocities during compatible prime conditions.

To explore this possibility, we used a modified version of Woestenburg et al. (1983) orthogonal polynomial trend analysis (OPTA; for a detailed description of this analysis, see Finkbeiner et al., 2013, and Karayanidis et al., 2011). Trials with correct responses in each individual cell of the experimental design (i.e., compatible and incompatible trials) were ranked according to their MIT. The trial with the fastest MIT was assigned the covariate value “1,” the next fastest a “2,” all the way up to the trial with the slowest MIT, which was assigned the covariate value “N,” corresponding to the total number of correct trials in that cell of the design. Next, a polynomial regression model was fitted to the x-velocities using MIT rank as the covariate and polynomial terms up to the 6th order. Terms that failed to explain significant variance were removed and the remaining coefficients were then used to generate *predicted* velocity profiles, one for each trial for each subject. To visualize the effect of target-viewing time on reaching responses, predicted trajectories were averaged into semi-decile intervals, resulting in 10 predicted trajectories per experimental condition, per subject. The first of these “percentiles” represents those trials corresponding to the fastest 10% of MIT latencies; the second represents the next fastest 10% MITs, and so forth. This was done first within subjects and then across subjects to obtain a group mean. For statistical analyses, we computed the mean x-velocity value from the first 50 samples of the predicted trajectories (i.e., the first 50% of the trajectory), which reduced each trial to a single value referred to as *initial* *x-velocity*. By limiting our dependent measure to the initial portion of the reaching trajectory, we were able to quantify how much the subject knew about the target at the time of movement initiation. Once compiled, initial x-velocity values were analyzed across all levels of prime-target compatibility (compatible vs. incompatible) and MIT percentile (1 through to 10) using a repeated-measures ANOVA. The OPTA procedure was implemented using custom software written in R ().

**FIGURE 4:**
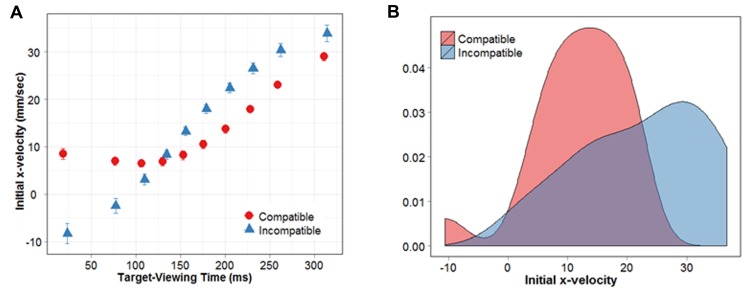
**(A)** Initial x-velocity shown as a function of target-viewing time. **(B)** Distribution of initial x-velocities for MIT percentiles 6–8.

The repeated-measures ANOVA revealed a significant main effect of MIT percentile (*F*_(1,9)_ = 48.78, *p* < 0.01), and a significant interaction between MIT percentile and prime-target compatibility (*F*_(1,9)_ = 8.97, *p* < 0.01). **Figure 4A** clearly depicts the main effect of MIT percentile, in that the initial x-velocity of reaching responses increases with longer MITs. In other words, the longer subjects take to view the target before they initiate their response, the faster their finger travels in the correct direction.

To examine the nature of the interaction, we conducted a series of paired *t*-tests (FDR-corrected) between compatible and incompatible conditions, at each MIT percentile. Results revealed that, for MIT percentiles 1, 2 and 3, the initial x-velocity of responses during compatible conditions was significantly greater compared with incompatible conditions (*p* = 0.003, 0.003, 0.03). This compatibility effect was reversed for percentiles 6, 7 and 8, where initial x-velocities were significantly greater during incompatible compared with compatible conditions (*p* = 0.02, 0.01, 0.003). As demonstrated in **Figure 4A**, movements initiated within the first 3 MIT percentiles (first 30% of the movement initiation distribution) were directly influenced by the prime arrow. Specifically, they moved in the correct direction (positive x-velocity) when the upcoming target was compatible, and in the incorrect direction when it was incompatible (negative x-velocity). When subjects had more time to view the target stimulus before beginning their movement (i.e., MIT percentiles 6–8), they were better at classifying the target arrow when it followed an *incompatible* prime. For simplicity, we will refer to MIT percentiles 6–8 as “NCE percentiles,” given that they show response costs during compatible prime conditions, and response benefits during incompatible prime conditions. Interestingly, within our NCE percentiles subjects’ responses did not exhibit a net movement in the wrong direction. Even though initial x-velocity was slower for compatible versus incompatible trials, it remained positive (i.e., in the correct direction) in all percentiles exhibiting an NCE.

The results reported above suggest that perceptual interactions between the prime and mask did not activate the “incorrect” response relative to the target. However, it was necessary to establish that our NCE percentiles were not comprised of a greater proportion of negative x-velocity trials in the compatible condition compared with the incompatible condition. For example, the NCE observed in these percentiles could be driven by (a) smaller x-velocity values in the compatible condition, or (b) a larger percentage of negative x-velocity trials. The former would indicate that the correct response had been inhibited because subjects simply take longer to move in the correct direction, whereas the latter would suggest that the incorrect response had been selectively activated on some trials. To distinguish between these two possibilities, we examined the distribution of x-velocities in the NCE percentiles. The results of our FDR corrected *t*-tests were used to separate the data into two subsets: one PCE subset (corresponding to MIT percentiles 1, 2, and 3) and one NCE subset (corresponding to MIT percentiles 6, 7 and 8). Should a larger proportion of negative x-velocity trials drive the NCE in the compatible condition, the distribution of initial x-velocities in our NCE percentiles should be *bimodal*. This is because while most x-velocities will be positive (we know this because average x-velocity is always positive), a proportion of trials should have x-velocity values that fall below 0. On the other hand, if the distribution of x-velocities is *unimodal*, it would indicate that the difference between compatible and incompatible conditions is being driven by smaller x-velocity values in the compatible condition. **Figure 4B** shows the distribution of initial x-velocities for our NCE percentiles. Hartigans’ dip test, which is a test for multimodality, failed to reveal any evidence for multimodality in either the compatible or incompatible distributions, suggesting they were both unimodal (*D* = 0.0864, *p* = 0.2646; *D* = 0.0676, *p* = 0.6953, respectively). These results suggest that the NCE we have observed is due to smaller x-velocities overall, not a mixture of positive and negative x-velocities (i.e., movements in the incorrect direction).

### PRIME DETECTION

The test of prime visibility revealed that our masking procedure was effective in preventing visual awareness of the prime stimuli. On average, subjects’ ability to correctly classify the prime was at chance (50% hit rate). This yielded a *d*’ score of 0.008, which was not significantly different from 0 (*p* = 0.82).

## DISCUSSION

There is an ongoing debate in the literature regarding the nature of the NCE. Of particular interest to us was whether, as predicted by the mask-induced priming account, the NCE is indeed driven by the activation of the opposite-to-prime, incorrect response when prime and mask stimuli share perceptual features. We combined a continuous behavioral measure with a response signal technique that allowed us to obtain an index of *when* positive and negative compatibility effects emerge, and *how* they manifest in behavior. LRP findings suggest that the modulatory effects of the prime stimulus on behavior should occur within two distinct and sequential phases following prime onset (Eimer and Schlaghecken, 1998). The first of these is an activation of the prime-induced motor response, and the second is characterized by its inhibition. We thus elicited reach-to-touch responses across a wide set of target-viewing times under the premise that positive and negative compatibility effects would emerge naturally depending on which stage (prime-activation or prime-inhibition) response initiation began. We report two key findings relating to the impact of unconscious information processing on behavior, which taken together encourage us to question existing conceptualisations regarding the mechanisms underlying the NCE.

Firstly, our pathoffset analysis confirmed that positive and negative compatibility effects could be elicited by varying target-viewing time. Responses initiated during the first “prime-activation” phase (i.e., pure prime trials) were characterized by a PCE, with smaller pathoffset values when the target was preceded by a response-compatible compared with a response-incompatible prime. Conversely, responses initiated during the second “prime-inhibition” phase (i.e., prime + target trials) had larger pathoffset values when targets were preceded by compatible compared with incompatible primes. These results are in line with previous behavioral studies that employed two prime-target SOAs to elicit PCEs and NCEs, and demonstrate that the emergence of these effects is driven by target-viewing time rather than specific experimental conditions.

In a separate analysis, we used velocity in the task-relevant (left/right) dimension to ascertain whether subjects’ response certainty during the first portion of their reaching trajectory was related to the amount of time they had spent viewing the target prior to movement onset. Consistent with our pathoffset results, this analysis revealed an interaction between target-viewing time and subjects’ ability to correctly classify the target arrow. When reaching movements were initiated very close to target onset, we found evidence of a PCE such that initial x-velocity was negative (i.e., in the incorrect direction) when the prime was incompatible, and positive (i.e., in the correct direction) when the prime was compatible. Longer target-viewing times resulted in an NCE, where subjects were faster at correctly classifying the target when it was preceded by an incompatible compared with a compatible prime. These findings are consistent with previous studies that examined the temporal dynamics of positive and negative compatibility effects using response-distribution analyses. This method involves ordering button-press responses from fast to slow, and then analysing the resulting response-time distribution by deciles. Using this approach, it has been shown that slow responses are typically characterized by an NCE, whereas faster responses exhibit a PCE (Eimer, 1999). Furthermore, when prime-target SOA is explicitly manipulated, the NCE becomes larger as the SOA is increased from 0 to 96 ms (Schlaghecken and Eimer, 1997, 2000; Aron et al., 2003). Our findings extend upon this previous work by revealing the analog unfolding of positive and negative compatibility effects as they occur in stimulus processing time, all within a single experimental context.

More importantly, our results provide behavioral evidence that in situations where the prime and mask are comprised of common features, the NCE does not appear to be driven by an *activation* of the incorrect response. If, as put forward by Lleras and Enns (2004), the NCE in our study was produced by a newly emergent percept resembling an opposite-to-prime arrow, we should have observed one of two possible scenarios. The first is that in the compatible condition, the initial direction of movements should have been made in the wrong direction. However, our results clearly show that while subjects were slower to classify the target arrow during compatible compared with incompatible conditions, reaching movements were always in the correct direction. The second possibility is based on the principle that prime-mask interactions may not *always* interact in such a way as to produce a new “opposite-prime.” In this case, the NCE would be driven by a subset of trials in which subjects did indeed misperceive the prime, and thus moved in the incorrect direction. To address this possibility we analyzed the distribution of initial x-velocities. Recall that x-velocity values are positive when the subject moves in the correct direction and negative when they move in the wrong direction. We found that x-velocities in the compatible condition were generally positive and formed a unimodal distribution, confirming that our NCE overall was driven by slower movements in the correct direction rather than a mixture of movements in the correct and incorrect direction. Our findings therefore challenge the accepted view that when a prime and mask share perceptual features, the NCE is driven by mask-induced activations of the opposite response. Instead, we provide evidence in line with the self-inhibition account of the NCE, which posits that the prime-activated response is automatically inhibited at long prime-target SOAs.

It is worth mentioning that the ideomotor principle that perception and action are inextricably linked can provide an alternative account for the present results. It has been shown that information processing can “work backwards” from action to perception, such that planning and executing a goal-directed action can impair the concurrent perception of action-compatible stimuli (Musseler and Hommel, 1997a,b). According to Hommel et al.’s (2001) two-phase model, making a left keypress in response to, say, the word “left” requires the initial *activation* of a so-called “feature code” (in this case, LEFT), and the subsequent *integration* of that code into a coherent action plan. Importantly, the second “integration phase” is also characterized by the self-inhibition of the feature code LEFT below baseline activity until the appropriate action is carried out. As such, this code would become temporarily unavailable for coding purposes, so that “left” objects (e.g., a left-pointing arrow) would be more difficult to code than “right” objects. This model predicts that during the activation phase, feature overlap between action plans and perceptual objects should yield positive priming effects because an already activated feature code should facilitate feature coding. Once a code has been *integrated*, however, it becomes “occupied” and should thus result in an impaired ability to interpret perceptually similar objects.

If we were to treat our prime and target arrows as two perceptually similar objects that share a common code (i.e., as being ideomotor compatible), this theory would predict the results of the present study. In this scenario, the prime stimulus (e.g., left-pointing arrow) would activate a relevant feature code, which includes a propensity for “leftward” action. If a response to a subsequent, left arrow target is initiated during this initial feature-*activation* phase, PCEs should occur. Responses initiated later on in time, however, might occur during the second phase wherein the feature code activated by the prime has been *integrated*. At this point the “LEFT” code has been occupied and responses to left arrow targets should be impeded (the NCE). Although this interpretation is feasible, the issue of conscious awareness is problematic. Stoet and Hommel (1999) argued that the integration phase is likely to be associated with the specific preparation of a particular action. That is, performance costs during the classification of perceptually relevant stimuli should only occur when such stimuli appear while an action is being *intentionally* planned and held in readiness (or while it is being executed). But in the present study, participants could not consciously perceive the prime arrow. It is therefore questionable whether the prime would have triggered a specific plan to execute its associated motor response, leading to the occupation of its associated feature code. Nevertheless, this is an interesting new perspective from which to consider the present findings and the NCE in general.

Traditionally, on-line control processes responsible for terminating incorrectly activated response programs were assumed to be (at least) highly correlated with consciousness and effort (Norman and Shallice, 1986; Johnson and Proctor, 2004). The NCE has challenged this widely held view by suggesting that inhibitory control can occur on the basis of bottom-up sensory processing. Mask-induced priming accounts have offered an alternative explanation for the effect using an established response priming model that does not recruit an off-line inhibitory mechanism. In the present experiment we were able to document the unfolding influence of masked prime stimuli on behavior. Contrary to the predictions made by mask-induced priming, the NCE was not driven by a tendency to move in the incorrect direction following the presentation of a compatible prime. We therefore provide evidence in support of emerging views that challenge the traditional dichotomy between low-level “automatic” processes on the one hand, and high-level “control” processes on the other (Wegner, 2002; Aarts et al., 2005; Hommel, 2007). Although our findings cannot adjudicate between other hypotheses put forth regarding the nature of the NCE (e.g., the mask-triggered inhibition hypothesis put forward by Jaskowski and Przekoracka-Krawczyk, 2005), they nevertheless prompt a further exploration of the possibility that low-level self-inhibition processes may be involved even in situations of prime/mask relevance.

## CONCLUSION

The results reported here firmly establish that the non-conscious processing of a prime arrow can yield two distinct congruency effects within the same experimental context. Responding via a reaching movement allows subjects to *initiate* their categorisation response very early in stimulus processing without penalty, thereby allowing the researcher to observe experimental effects as they emerge in stimulus processing time. Using this technique we were able to show positive and negative compatibility effects as a function of target-viewing time. Crucially, our reach-to-touch paradigm allowed us to determine that the NCE was not produced by movements in the incorrect direction but, rather, by movements that traveled in the correct direction more slowly. This is consistent with the notion of an automatic inhibitory mechanism whose role is to dampen motor activations elicited by stimuli that are no longer supported by perceptual input (Schlaghecken et al., 2007).

## AUTHOR CONTRIBUTIONS

Conceived and designed the experiments: Brenda Ocampo, Matthew Finkbeiner. Performed the experiments: Brenda Ocampo. Analyzed the data: Brenda Ocampo, Matthew Finkbeiner. Contributed reagents/materials/analysis tools: Matthew Finkbeiner. Wrote the paper: Brenda Ocampo, Matthew Finkbeiner.

## Conflict of Interest Statement

The authors declare that the research was conducted in the absence of any commercial or financial relationships that could be construed as a potential conflict of interest.

## References

[B1] Aarts H. Custers R. Wegner D. M. (2005). On the inference of personal authorship: enhancing experienced agency by priming effect information. *Conscious. Cogn.* 14 439–458 10.1016/j.concog.2004.11.001 16091264

[B2] Ansorge U. Klotz W. Neumann O. (1998). Manual and verbal responses to completely masked (unreportable) stimuli: exploring some conditions for the metacontrast dissociation. *Perception* 27 1177–1189 10.1068/P271177 10505196

[B3] Aron A. R. Schlaghecken F. Fletcher P. C. Bullmore E. T. Eimer M. Barker R. (2003). Inhibition of subliminally primed responses is mediated by the caudate and thalamus: evidence from functional MRI and Huntington’s disease. *Brain* 126 713–723 10.1093/Brain/Awg067 12566291 PMC3838934

[B4] Chapman C. S. Gallivan J. P. Wood D. K. Milne J. L. Culham J. C. Goodale M. A. (2010). Reaching for the unknown: multiple target encoding and real-time decision-making in a rapid reach task. *Cognition* 116 168–176 10.1016/j.cognition.2010.04.008 20471007

[B5] Dale R. Kehoe C. Spivey M. J. (2007). Graded motor responses in the time course of categorizing atypical exemplars. *Mem. Cognit.* 35 15–28 10.3758/BF03195938 17533876

[B6] Damian M. F. (2001). Congruity effects evoked by subliminally presented primes: automaticity rather than semantic processing. *J. Exp. Psychol. Hum. Percept. Perform.* 27 154–165 10.1037/0096-1523.27.1.154 11248930

[B7] Egner T. (2010). Motor control: exploring the neurochemistry of subliminal inhibition. *Curr. Biol.* 20 R852–R853 10.1016/j.cub.2010.08.051 20937473

[B8] Eimer M. (1999). Facilitatory and inhibitory effects of masked prime stimuli on motor activation and behavioural performance. *Acta. Psychol.* 101 293–313 10.1016/S0001-6918(99)00009-8 10344189

[B9] Eimer M. Schlaghecken F. (1998). Effects of masked stimuli on motor activation: behavioral and electrophysiological evidence. *J. Exp. Psychol. Hum. Percept. Perform.* 24 1737–1747 10.1037/0096-1523.24.6.1737 9861720

[B10] Eimer M. Schlaghecken F. (2003). Response facilitation and inhibition in subliminal priming. *Biol. Psychol.* 64 7–26 10.1016/S0301-0511(03)00100-5 14602353

[B11] Finkbeiner M. Coltheart M. Coltheart V. (2013). Pointing the way to new constraints on the dynamical claims of computational models. *J. Exp. Psychol. Hum. Percept. Perform.* 10.1037/a0033169 [Epub ahead of print]. 23750962

[B12] Finkbeiner M. Friedman J. (2011). The flexibility of nonconsciously deployed cognitive processes: evidence from masked congruence priming. *PLoS ONE *6:e17095. 10.1371/journal.pone.0017095 PMC3037407 21347336

[B13] Hommel B. (2007). Consciousness and control – not identical twins. *J. Conscious. Stud.* 14 155–176

[B14] Hommel B. Musseler J. Aschersleben G. Prinz W. (2001). The theory of event coding (TEC): a framework for perception and action planning. *Behav. Brain Sci.* 24 849–878 10.1017/S0140525X01000103 12239891

[B15] Jaskowski P. (2008). The negative compatibility effect with nonmasking flankers: a case for mask-triggered inhibition hypothesis. *Conscious. Cogn.* 17 765–777 10.1016/j.concog.2007.12.002 18226925

[B16] Jaskowski P. Przekoracka-Krawczyk A. (2005). On the role of mask structure in subliminal priming. *Acta Neurobiol. Exp.* 65 409–417 10.55782/ane-2005-1569 16366393

[B17] Jaskowski P. Van Der Lubbe R. H. J. Schlotterbeck E. Verleger R. (2002). Traces left on visual selective attention by stimuli that are not consciously identified. *Psychol. Sci.* 13 48–54 10.1111/1467-9280.00408 11894850

[B18] Johnson A. Proctor R. W. (2004). *Attention: Theory and Practice*. Thousand Oaks: Sage

[B19] Karayanidis F. Provost A. Brown S. Paton B. Heathcote A. (2011). Switch-specific and general preparation map onto different ERP components in a task-switching paradigm. *Psychophysiology* 48 559–568 10.1111/j.1469-8986.2010.01115.x 20718932

[B20] Kiefer M. Spitzer M. (2000). Time course of conscious and unconscious semantic brain activation. *Neuroreport* 11 2401–2407 10.1097/00001756-200008030-00013 10943693

[B21] Klapp S. T. (2005). Two versions of the negative compatibility effect: comment on Lleras and Enns (2004). *J. Exp. Psychol. Gen.* 134 431–435 10.1037/0096-3445.134.3.431 16131274

[B22] Klapp S. T. Hinkley L. B. (2002). The negative compatibility effect: unconscious inhibition influences reaction time and response selection. *J. Exp. Psychol. Gen.* 131 255–269 10.1037//0096-3445.131.2.255 12049243

[B23] Klinger M. R. Burton P. C. Pitts G. S. (2000). Mechanisms of unconscious priming: I. Response competition, not spreading activation. * J. Exp. Psychol. Learn. Mem. Cogn.* 26 441–455 10.1037//0278-7393.26.2.441 10764105

[B24] Leuthold H. Kopp B. (1998). Mechanisms of priming by masked stimuli: inferences from event-related brain potentials. *Psychol. Sci.* 9 263–269 10.1111/1467-9280.00053

[B25] Lleras A. Enns J. T. (2004). Negative compatibility or object updating? A cautionary tale of mask-dependent priming. *J. Exp. Psychol. Gen.* 133 475–493 10.1037/0096-3445.133.4.475 15584802

[B26] Lleras A. Enns J. T. (2006). How much like a target can a mask be? Geometric, spatial, and temporal similarity in priming: a reply to Schlaghecken and Eimer (2006). *J. Exp. Psychol. Gen.* 135 495–500 10.1037/0096-3445.135.3.495 16846278

[B27] Musseler J. Hommel B. (1997a). Blindness to response-compatible stimuli. *J. Exp. Psychol. Hum. Percept. Perform.* 23 861–872 10.1037/0096-1523.23.3.861 9180047

[B28] Musseler J. Hommel B. (1997b). Detecting and identifying response-compatible stimuli. *Psychon. Bull. Rev.* 4 125–129 10.3758/Bf03210785

[B29] Neumann O. Klotz W. (1994). “Motor-responses to nonreportable, masked stimuli – where is the limit of direct parameter specification,” in *Attention and Performance Xv* eds Umiltà C. Moscovitch M. (Denver: A Bradford Book) 15 123–150

[B30] Norman D. Shallice T. (1986). “Attention to action: willed and automatic control of behavior,” in *Consciousness and Self-Regulation*: *Advances in Research and Theory IV* eds Davidson R. Schwartz R. Shapiro D. (New York: Plenum Press) 1–18

[B31] Praamstra P. Seiss E. (2005). The neurophysiology of response competition: motor cortex activation and inhibition following subliminal response priming. *J. Cogn. Neurosci.* 17 483–493 10.1162/0898929053279513 15814007

[B32] Quek G. L. Finkbeiner M. (2013). Spatial and temporal attention modulate the early stages of face processing: behavioural evidence from a reaching paradigm. *PLoS ONE *8:e57365. 10.1371/journal.pone.0057365 PMC3585364 23468977

[B33] Schlaghecken F. Eimer M. (1997). The influence of subliminally presented primes on response preparation. *Sprache Kognition* 16 166–175

[B34] Schlaghecken F. Eimer M. (2000). A central-peripheral asymmetry in masked priming. *Percept. Psychophys.* 62 1367–1382 10.3758/Bf03212139 11143449

[B35] Schlaghecken F. Eimer M. (2001). Partial response activation to masked primes is not dependent on response readiness. *Percept. Mot. Skills* 92 208–222 10.2466/Pms.92.1.208-222 11322588

[B36] Schlaghecken F. Eimer M. (2002). Motor activation with and without inhibition: evidence for a threshold mechanism in motor control. *Percept. Psychophys.* 64 148–162 10.3758/Bf03194564 11916298

[B37] Schlaghecken F. Eimer M. (2006). Active masks and active inhibition: a comment on Lleras and Enns (2004) and on Verleger, Jaskowski, Aydemir, van der Lubbe, and Groen (2004). *J. Exp. Psychol. Gen.* 135 484–494 10.1037/0096-3445.135.5.484 16846277

[B38] Schlaghecken F. Rowley L. Sembi S. Simmons R. Whitcomb D. (2007). The negative compatibility effect: a case for self-inhibition. *Adv. Cogn. Psychol.* 3 227–240 10.2478/v10053-008-0027-y 20517511 PMC2864980

[B39] Schmidt T. (2002). The finger in flight: real-time motor control by visually masked color stimuli. *Psychol. Sci.* 13 112–118 10.1111/1467-9280.00421 11933993

[B40] Song J. H. Nakayama K. (2009). Hidden cognitive states revealed in choice reaching tasks. *Trends Cogn. Sci.* 13 360–366 10.1016/j.tics.2009.04.009 19647475

[B41] Spivey M. J. Grosjean M. Knoblich G. (2005). Continuous attraction toward phonological competitors. *Proc. Natl. Acad. Sci. U.S.A.* 102 10393–10398 10.1073/pnas.0503903102 15985550 PMC1177386

[B42] Stoet G. Hommel B. (1999). Action planning and the temporal binding of response codes. *J. Exp. Psychol. Hum. Percept. Perform.* 25 1625–1640 10.1037/0096-1523.25.6.1625

[B43] Verleger R. Jaskowski P. Aydemir A. Van Der Lubbe R. H. J. Groen M. (2004). Qualitative differences between conscious and nonconscious processing? On inverse priming induced by masked arrows. *J. Exp. Psychol. Gen.* 133 494–515 10.1037/0096-3445.133.4.494 15584803

[B44] Verleger R. Kotter T. Jaskowski P. Sprenger A. Siebner H. (2006). A TMS study on non-consciously triggered response tendencies in the motor cortex. *Exp. Brain Res.* 173 115–129 10.1007/s00221-006-0371-4 16506010

[B45] Wegner D. M. (2002). *The Illusion of Conscious Will*. Cambridge: Cambridge University Press

[B46] Woestenburg J. C. Verbaten M. N. Vanhees H. H. Slangen J. L. (1983). Single trial Erp estimation in the frequency-domain using orthogonal polynomial trend analysis (Opta) – estimation of individual habituation. *Biol. Psychol.* 17 173–191 10.1016/0301-0511(83)90018-2 6640015

